# Supplementary material for: Integrative multi-omics data provide insights into the biosynthesis of furanocoumarins and mechanisms regulating their accumulation in Angelica dahurica
Source: Commun Biol. 2025 Apr 23;8:649. doi: 10.1038/s42003-025-08076-x (PMC12019236; doi:10.1038/s42003-025-08076-x)
Supplement: Supplementary file 1 — Supplementary Information [file 42003_2025_8076_MOESM1_ESM.pdf]

# Supplemental information

## Integrative multi-omics data provide insights into the biosynthesis of furanocoumarins and mechanisms regulating their accumulation in *Angelica dahurica*

**Jiaojiao Ji[1#], Xiaoxu Han[1,2#], Lanlan Zang[1], Yushan Li[3], Liqun Lin[1], Donghua Hu[1,4], Shichao Sun[1], Yonglin Ren[2], Garth Maker[2], Zefu Lu[3*], Li Wang[1,5*]**

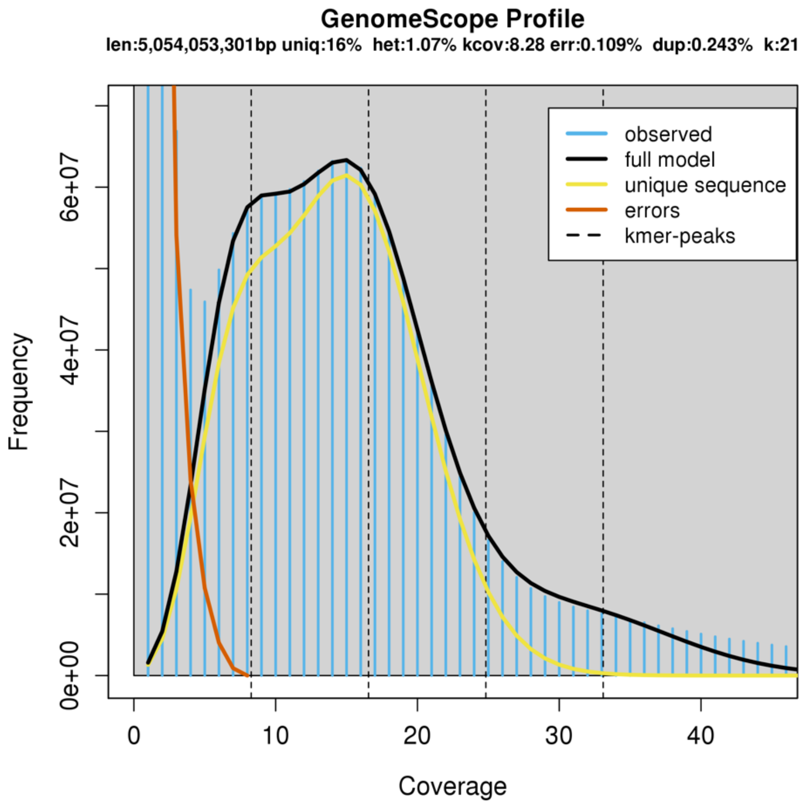


**Supplementary Figure 1** The K-mer depth distribution for genome size evaluation.

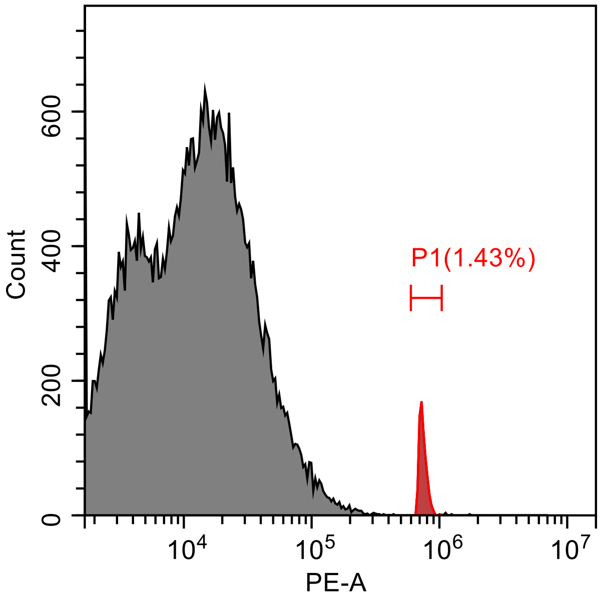


**Supplementary Figure 2** Flow cytometry with *Angelica sinensis* and *Foeniculum vulgare* as reference. Genome size was calculated as about 4.56 Gb.

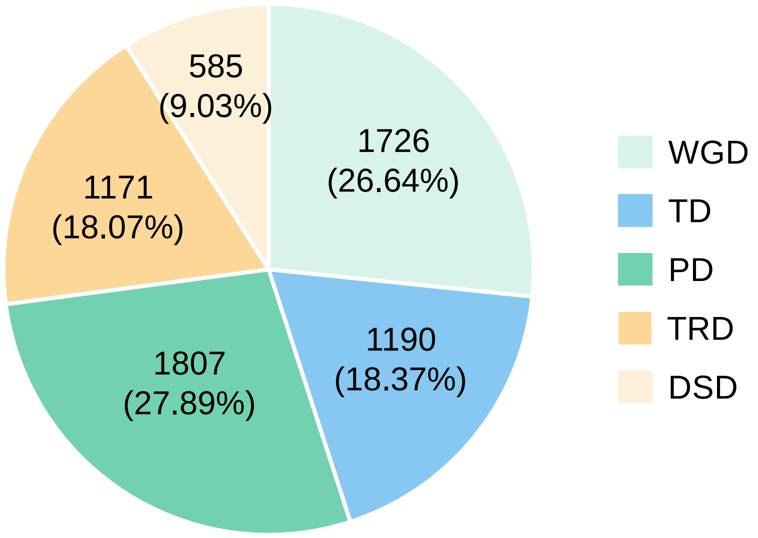


**Supplementary Figure 3** Classification of duplicated genes within expanded gene families in *Angelica dahurica*. WGD, whole-genome duplication, TD, tandem duplication, PD, proximal duplication, TRD, transposed duplication, DSD, dispersed duplication.

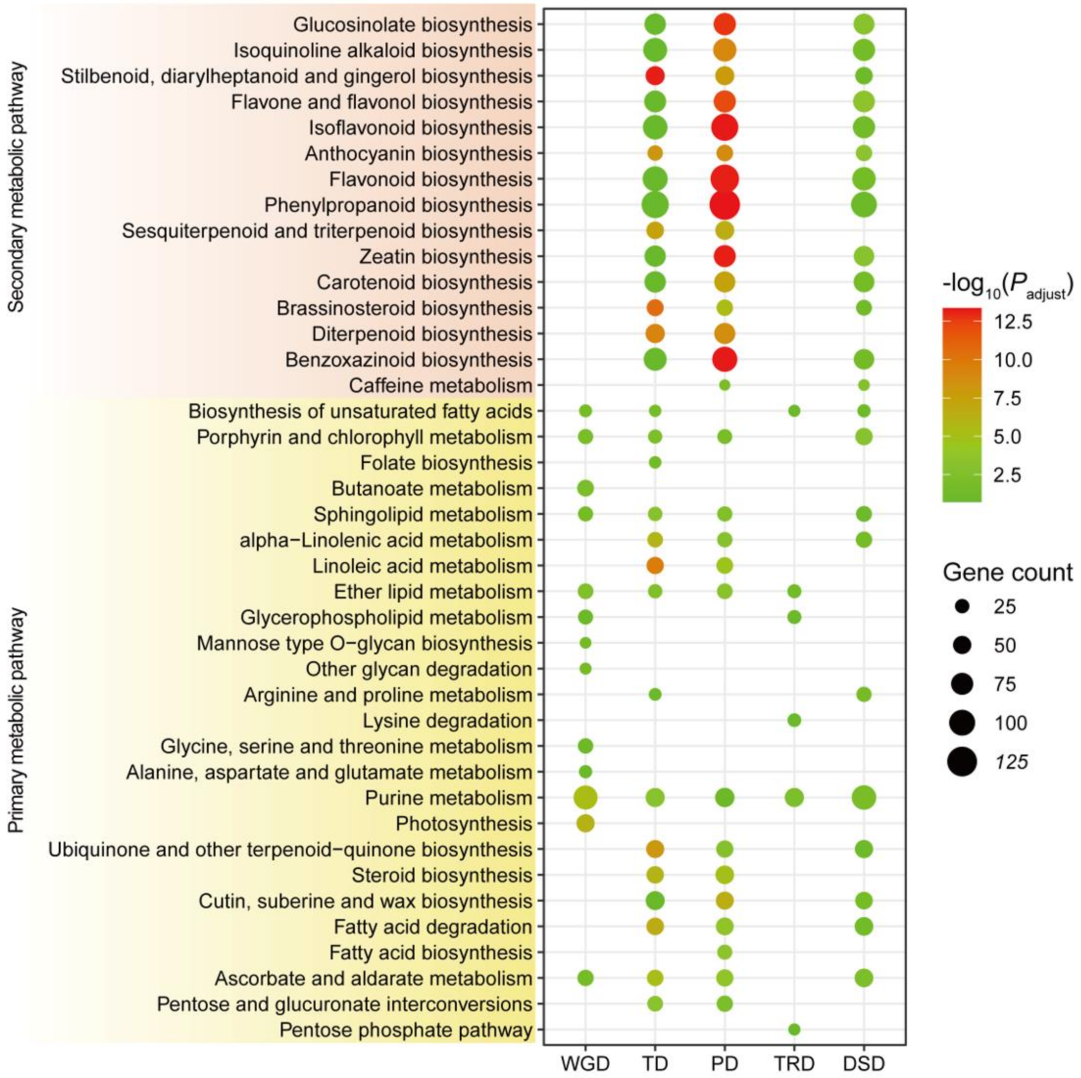


**Supplementary Figure 4** Kyoto Encyclopedia of Genes and Genomes (KEGG) enrichment analyses of different types of duplicated genes within expanded gene families. The enriched terms with adjusted $P < 0.05$ are presented. Color of the bubbles indicates statistical significance of the enriched terms; size of the bubbles indicates number of genes within the term.

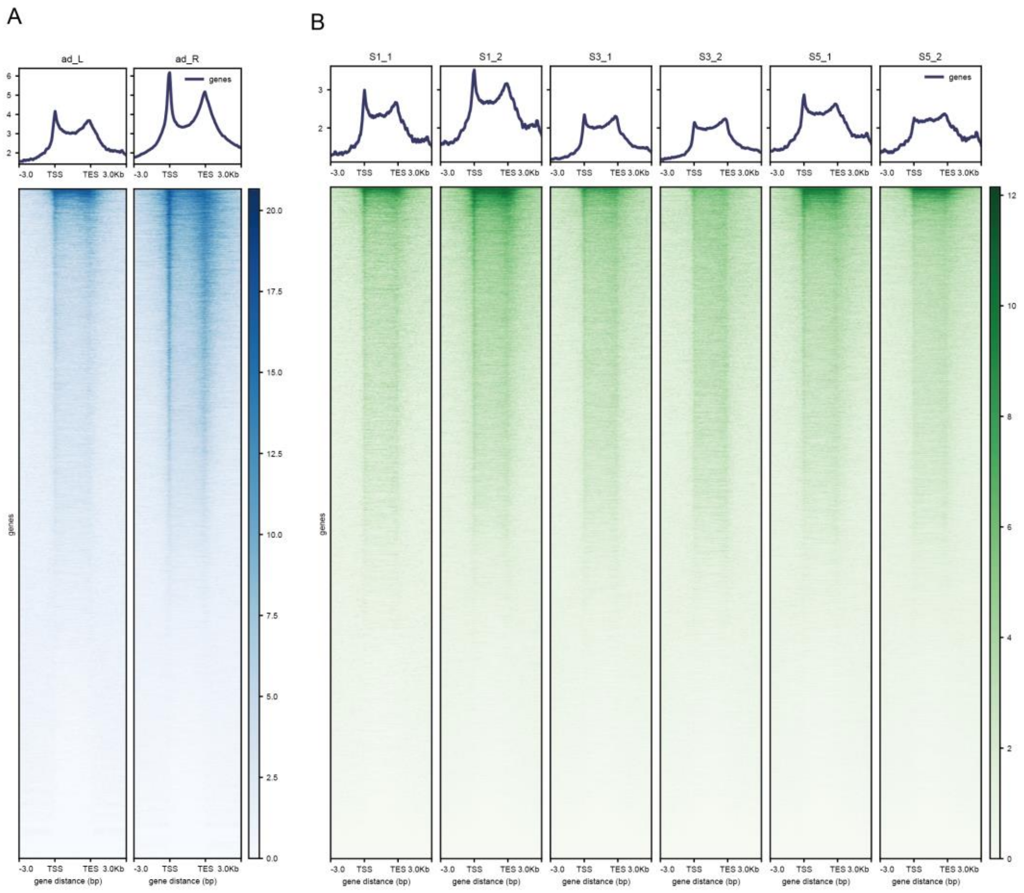


**Supplementary Figure 5** Chromatin accessibility profiles in gene regions. The 3 kb upstream and downstream flanking coding regions were aligned for all genes. TSS, transcription start site; TES, transcription end site.

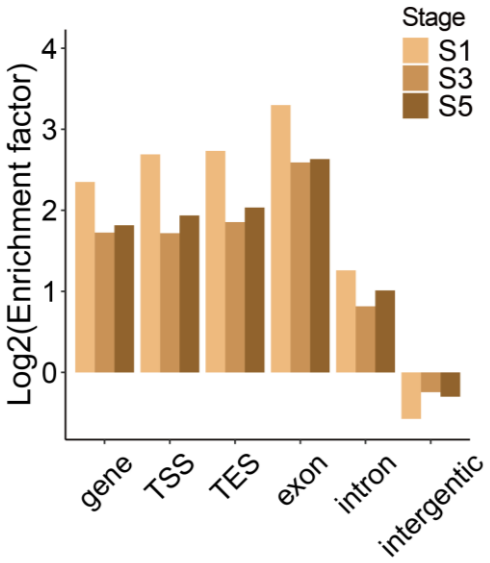


**Supplementary Figure 6** Bar plot of enrichment factor of peaks (ACRs) in gene, TSS (within 1 kb upstream and downstream of the TSS), TES (within 1 kb upstream and downstream of the TES), exon, intron, and intergenic regions.

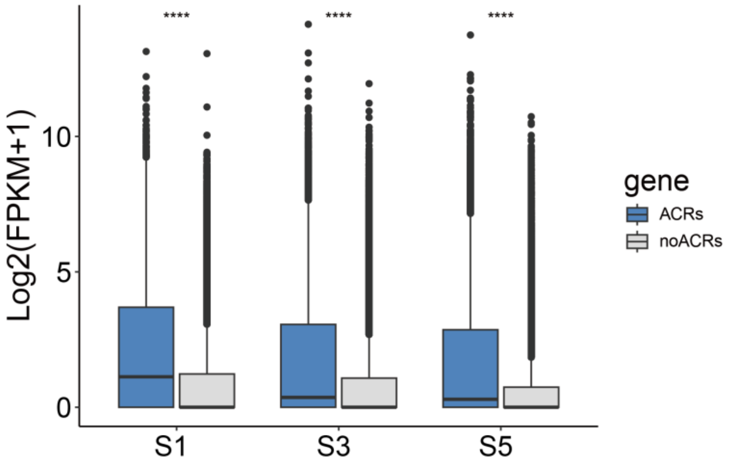


**Supplementary Figure 7** Expression levels of genes with or without related accessible regions (ACRs) in root development.

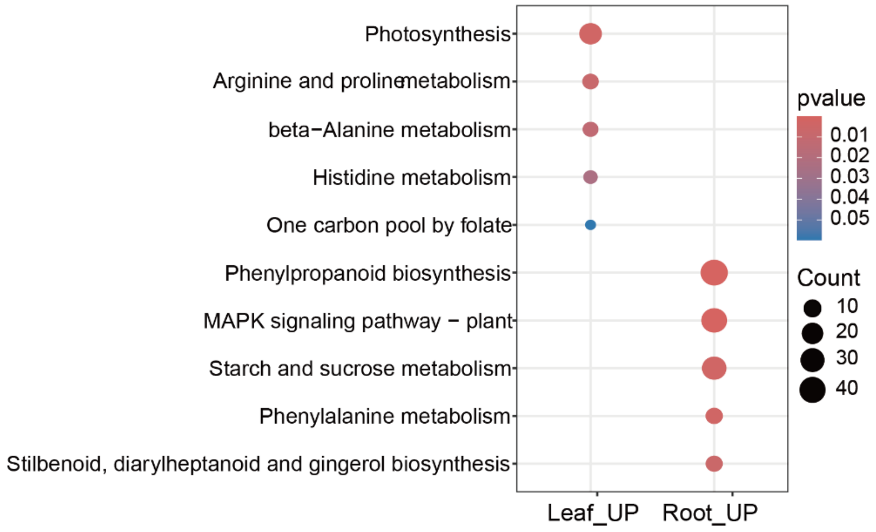


**Supplementary Figure 8** KEGG enrichment analysis of differential accessible regions (DARs) up-regulated in leaves (left) and root (right) of *Angelica dahurica***.**

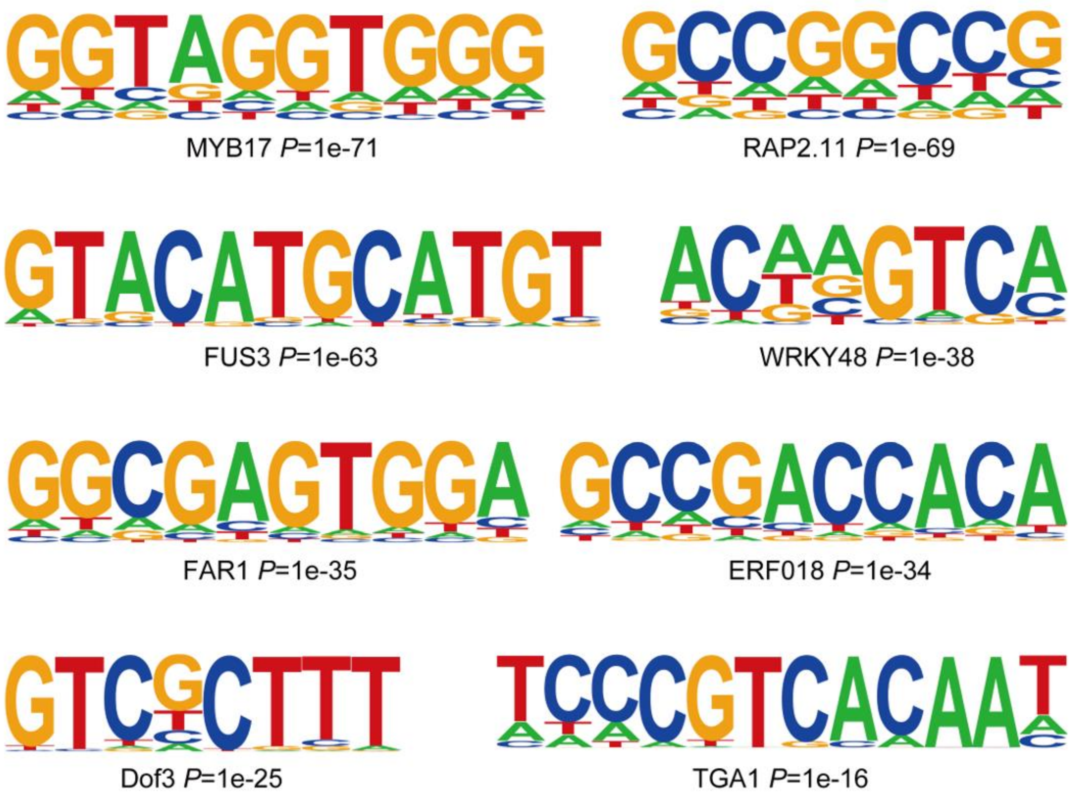


**Supplementary Figure 9** Motifs potentially regulating FC biosynthesis, enriched in DARs that are upregulated in the roots of *Angelica dahurica* relative to the leaves.

**Supplementary Table 1** Sequences generated in this study.

| | | | Base (bp) | Depth (×) |
|---|---|---|---|---|
| DNA | PacBio | | 85,862,285,723 | ~18 |
| | Hi-C | | 328,926,579,300 | ~70 |
| RNA | Illumina | Root_rep1 | 52,392,198 | ~11 |
| | | Root_rep2 | 54,644,086 | ~11 |
| | | Root_rep3 | 50,262,761 | ~10 |
| | | Flower_rep1 | 56,513,981 | ~11 |
| | | Flower_rep2 | 59,591,079 | ~12 |
| | | Flower_rep3 | 50,262,761 | ~10 |
| | | Stem_rep1 | 54,299,236 | ~11 |
| | | Stem_rep2 | 58,481,599 | ~12 |
| | | Stem_rep3 | 49,586,093 | ~10 |
| | | Mature_leaf_rep1 | 54,406,096 | ~11 |
| | | Mature_leaf_rep2 | 52,984,058 | ~11 |
| | | Mature_leaf_rep3 | 56,603,962 | ~12 |
| | | Young_leaf_rep1 | 53,074,317 | ~11 |
| | | Young_leaf_rep2 | 50,437,349 | ~10 |
| | | Young_leaf_rep3 | 54,613,159 | ~11 |
| | | S1_rep1 | 48,305,330 | ~10 |
| | | S1_rep2 | 54,633,251 | ~11 |
| | | S1_rep3 | 53,632,578 | ~11 |
| | | S2_rep1 | 43,101,499 | ~9 |
| | | S2_rep2 | 48,832,901 | ~10 |
| | | S2_rep3 | 45,668,244 | ~9 |
| | | S3_rep1 | 49,919,918 | ~10 |
| | | S3_rep2 | 48,329,470 | ~9 |
| | | S3_rep3 | 51,288,449 | ~11 |
| | | S4_rep1 | 49,779,770 | ~10 |
| | | S4_rep2 | 53,141,543 | ~11 |
| | | S4_rep3 | 49,630,061 | ~10 |
| | | S5_rep1 | 49,698,208 | ~10 |
| | | S5_rep2 | 52,724,835 | ~11 |
| | | S5_rep3 | 50,135,167 | ~10 |
| | | S6_rep1 | 52,548,098 | ~11 |
| | | S6_rep2 | 51,440,650 | ~11 |
| | | S6_rep3 | 52,891,402 | ~11 |
| ATAC | Illumina | Root | 39,340,836 | ~9 |
| | | Leaf | 53,992,304 | ~11 |
| | | S1_rep1 | 33,540,816 | ~7 |
| | | S1_rep2 | 88,318,468 | ~18 |
| | | S3_rep1 | 62,477,708 | ~13 |
| | | S3_rep2 | 61,167,926 | ~13 |

| | | | | |
|---|---|---|---|---|
| | | **S5_rep1** | 73,942,922 | ~15 |
| | | **S5_rep2** | 117,139,522 | ~24 |

**Supplementary Table 2** Assembly and annotation statistics of the *Angelica dahurica* genome.

| | |
|---|---|
| **Total assembly size (bp)** | 4897920425 |
| **Total scaffold number** | 146 |
| **Maximum scaffold length (bp)** | 506786926 |
| **Scaffold N50 (bp)** | 472440809 |
| **Scaffold N90 (bp)** | 353591046 |
| **GC content (%)** | 35.60 |
| **BUSCO# (%)** | 97.20 |
| **LAI** | 20.81 |

#BUSCO anotation using viridiplantae_odb10

**Supplementary Table 3** The mapping rates of *Angelica dahurica* in different tissues.

| Tissue | Read Numbers | Mapping Rate |
|---|---|---|
| S1-1 | 48,305,330 | 95.36% |
| S1-2 | 54,633,251 | 95.02% |
| S1-3 | 53,632,578 | 95.44% |
| S2-1 | 43,101,499 | 94.17% |
| S2-2 | 48,832,901 | 93.91% |
| S2-3 | 45,668,244 | 93.90% |
| S3-1 | 49,919,918 | 95.41% |
| S3-2 | 48,329,470 | 95.49% |
| S3-3 | 51,288,449 | 95.61% |
| S4-1 | 49,779,770 | 95.56% |
| S4-2 | 53,141,543 | 95.62% |
| S4-3 | 49,630,061 | 95.63% |
| S5-1 | 49,698,208 | 95.47% |
| S5-2 | 52,724,835 | 95.15% |
| S5-3 | 50,135,167 | 95.29% |
| S6-1 | 52,548,098 | 94.68% |
| S6-2 | 51,440,650 | 95.23% |
| S6-3 | 52,891,402 | 95.62% |
| R-1 | 52,392,198 | 95.30% |
| R-2 | 54,644,086 | 94.81% |
| R-3 | 50,262,761 | 96.01% |
| S-1 | 54,299,236 | 95.51% |
| S-2 | 58,481,599 | 96.15% |
| S-3 | 49,586,093 | 95.44% |
| ML-1 | 54,406,096 | 83.84% |
| ML-2 | 52,984,058 | 81.51% |
| ML-3 | 56,603,962 | 84.29% |
| YL-1 | 53,074,317 | 86.66% |
| YL-2 | 50,437,349 | 91.48% |
| YL-3 | 54,613,159 | 92.73% |
| F-1 | 56,513,981 | 95.84% |
| F-2 | 59,591,079 | 96.05% |
| F-3 | 50,262,761 | 96.01% |

**Supplementary Table 4** Statistics of *Angelica dahurica* pseudomolecules.

| **Chromosome** | **Total length (bp)** | **GC content (%)** | **Gene number** |
|---|---|---|---|
| Chromosome 1 | 506786926 | 35.67 | 6000 |
| Chromosome 2 | 501082715 | 35.69 | 6447 |
| Chromosome 3 | 490277005 | 35.65 | 5806 |
| Chromosome 4 | 479219616 | 35.61 | 5773 |
| Chromosome 5 | 472440809 | 35.75 | 6726 |
| Chromosome 6 | 432978375 | 35.7 | 5918 |
| Chromosome 7 | 401735355 | 35.75 | 4860 |
| Chromosome 8 | 400149488 | 35.69 | 4730 |
| Chromosome 9 | 390539985 | 35.75 | 5215 |
| Chromosome 10 | 353591046 | 35.69 | 5005 |
| Chromosome 11 | 335116718 | 35.76 | 4586 |
| Unmapped | 134002387 | 35.59 | 353 |
| Total | 4897920425 | 35.60 | 61419 |

**Supplementary Table 5** Annotation statistics of the *Angelica dahurica* genome.

| | |
|---|---|
| Gene number | 61419 |
| Average number of exons per gene | 3.93 |
| Total exon length (Mb) | 63921232 |
| Average exon length (bp) | 264.69 |
| Average number of introns per gene | 2.93 |
| Total intron length (Mb) | 109246157 |
| Average intron length (bp) | 606.68 |
| BUSCO[#] (%) | 91.00 |
| Number of genes annotated in NR | 53412 |
| Number of genes annotated in KEGG | 31799 |
| Number of genes annotated in Pfam | 35177 |
| Number of genes annotated in GO | 26042 |

#BUSCO anotation using viridiplantae_odb10

**Supplementary Table 6** Repeat contents in the *Angelica dahurica* genome.

| Class | Superfamily | Count | Length (bp) | Proportion |
|---|---|---|---|---|
| LTR | Copia | 1216361 | 1417643068 | 28.95% |
| LTR | Gypsy | 911300 | 1187106809 | 24.24% |
| LTR | unknown | 1560595 | 1195529872 | 24.41% |
| TIR | CACTA | 232178 | 97986548 | 2.50% |
| TIR | Mutator | 271674 | 104900806 | 1.40% |
| TIR | PIF_Harbinger | 59310 | 16859532 | 0.29% |
| TIR | Tc1_Mariner | 208479 | 63621194 | 0.82% |
| TIR | hAT | 168495 | 56472214 | 0.99% |
| nonTIR | helitron | 67893 | 23853899 | 3.47% |
| | Total | 4966673 | 4264156825 | 87.08% |
