# Supplementary material for: Integrative multi-omics data provide insights into the biosynthesis of furanocoumarins and mechanisms regulating their accumulation in Angelica dahurica
Source: Commun Biol. 2025 Apr 23;8:649. doi: 10.1038/s42003-025-08076-x (PMC12019236; doi:10.1038/s42003-025-08076-x)
Supplement: Supplementary file 2 — Description of Additional Supplementary Files [file 42003_2025_8076_MOESM2_ESM.docx]

Description of Additional Supplementary Files

**File Name:** Supplementary Data 1

**Description:** Gene expression level (FPKM) during root development of Angelica dahurica.

**File Name:** Supplementary Data 2

**Description:** Gene expression level (FPKM) of different tissues in Angelica dahurica.

**File Name:** Supplementary Data 3

**Description:** Pearson correlation between FPKM and metabolite levels in different tissues.

**File Name:** Supplementary Data 4

**Description:** Validated protein sequences in the FC biosynthetic pathway.

**File Name:** Supplementary Data 5

**Description:** The source data behind the graphs
