# Supplementary material for: Integrative multi-omics data provide insights into the biosynthesis of furanocoumarins and mechanisms regulating their accumulation in Angelica dahurica
Source: Commun Biol. 2025 Apr 23;8:649. doi: 10.1038/s42003-025-08076-x (PMC12019236; doi:10.1038/s42003-025-08076-x)
Supplement: Supplementary file 5 — Reporting Summary [file 42003_2025_8076_MOESM5_ESM.pdf]

nature portfolio

Corresponding author(s): Zefu Lu, Li Wang

Last updated by author(s): Mar 19, 2025

# Reporting Summary

Nature Portfolio wishes to improve the reproducibility of the work that we publish. This form provides structure for consistency and transparency in reporting. For further information on Nature Portfolio policies, see our Editorial Policies and the Editorial Policy Checklist.

## Statistics

For all statistical analyses, confirm that the following items are present in the figure legend, table legend, main text, or Methods section.

| n/a | Confirmed | |
|---|---|---|
| ☐ | ☒ | The exact sample size (*n*) for each experimental group/condition, given as a discrete number and unit of measurement |
| ☐ | ☒ | A statement on whether measurements were taken from distinct samples or whether the same sample was measured repeatedly |
| ☒ | ☐ | The statistical test(s) used AND whether they are one- or two-sided<br>*Only common tests should be described solely by name; describe more complex techniques in the Methods section.* |
| ☒ | ☐ | A description of all covariates tested |
| ☐ | ☒ | A description of any assumptions or corrections, such as tests of normality and adjustment for multiple comparisons |
| ☐ | ☒ | A full description of the statistical parameters including central tendency (e.g. means) or other basic estimates (e.g. regression coefficient) AND variation (e.g. standard deviation) or associated estimates of uncertainty (e.g. confidence intervals) |
| ☒ | ☐ | For null hypothesis testing, the test statistic (e.g. *F*, *t*, *r*) with confidence intervals, effect sizes, degrees of freedom and *P* value noted<br>*Give P values as exact values whenever suitable.* |
| ☒ | ☐ | For Bayesian analysis, information on the choice of priors and Markov chain Monte Carlo settings |
| ☒ | ☐ | For hierarchical and complex designs, identification of the appropriate level for tests and full reporting of outcomes |
| ☐ | ☒ | Estimates of effect sizes (e.g. Cohen's *d*, Pearson's *r*), indicating how they were calculated |

*Our web collection on statistics for biologists contains articles on many of the points above.*

## Software and code

Policy information about availability of computer code

| | |
|---|---|
| Data collection | No public data was involved in this study. |
| Data analysis | Genome size estimation: CytExpert software v.2.3, Jellyfish v2.3.0.<br>Genome assembly: Hifiasm v0.15.5-r350, Purge Haplotigs v1.1.1 61, Juicer v1.6, 3D-DNA v180114, Juicebox Assembly Tools v2.18 (https://github.com/aidenlab/Juicebox), Benchmarking Universal Single-Copy Orthologs (BUSCO v5.1.2) and LTR Assembly Index (LAI).<br>Genome annotion: EDTA v2.0.1, AUGUSTUS v.2.3.3, MAKER v3.01.03, GFAP, EGGNOG-MAPPER v.1.0.3, KEGG (https://www.kegg.jp), GO ( http://geneontology.org), PFAM (http://pfam.xfam.org/).<br>Phylogenetic analyses: OrthoFinder v2.5.2, MAFFT v7.271, trimAl v1.4.rev22, RAxML v.8.2.12, TIMETREE (http://www.timetree.org/), CAFE5 v5.0, MCscanX v1.1.11, BLASTP v2.10.0, DupGen_Finder v1.12, TBtools v2.030, One Step MCScanX of TBtools v2.154.<br>Transcriptome analyses: Hisat2 v2.2.1, Stringtie v2.1.4, DESeq2 v2_1.36.0, R package clusterProfiler v4.4.4.<br>ATAC-seq analysis: Fastp v0.12.4, Bowtie2 v2.4.2, Sambamba v0.8.0, SAMtools v1.13, deepTools v3.5.2, Integrative Genomics Viewer (IGV) v2.17.0, MACS2 v2.2.6, CHIPSEEKER v1.16.1. |

For manuscripts utilizing custom algorithms or software that are central to the research but not yet described in published literature, software must be made available to editors and reviewers. We strongly encourage code deposition in a community repository (e.g. GitHub). See the Nature Portfolio guidelines for submitting code & software for further information.

# Data

Policy information about availability of data

All manuscripts must include a data availability statement. This statement should provide the following information, where applicable:

- Accession codes, unique identifiers, or web links for publicly available datasets
- A description of any restrictions on data availability
- For clinical datasets or third party data, please ensure that the statement adheres to our policy

The data that support the findings of this study, including the raw genome, RNAseq and ATAC-seq data, were deposited in the CNGB Nucleotide Sequence Archive (https://db.cngb.org/cnsa) and are accessible with the accession ID CNP0005587. The assembled genome files are available at figshare (https://figshare.com/s/bea35667a2b27179a121).

# Research involving human participants, their data, or biological material

Policy information about studies with human participants or human data. See also policy information about sex, gender (identity/presentation), and sexual orientation and race, ethnicity and racism.

| | |
|---|---|
| Reporting on sex and gender | This study did not involve any human participants. |
| Reporting on race, ethnicity, or other socially relevant groupings | N/A |
| Population characteristics | N/A |
| Recruitment | N/A |
| Ethics oversight | N/A |

Note that full information on the approval of the study protocol must also be provided in the manuscript.

# Field-specific reporting

Please select the one below that is the best fit for your research. If you are not sure, read the appropriate sections before making your selection.

☒ Life sciences ☐ Behavioural & social sciences ☐ Ecological, evolutionary & environmental sciences

For a reference copy of the document with all sections, see nature.com/documents/nr-reporting-summary-flat.pdf

# Life sciences study design

All studies must disclose on these points even when the disclosure is negative.

| | |
|---|---|
| Sample size | The sample size was determined (minimum of two independent samples) based on different experiment to ensure similar results are reproducible. |
| Data exclusions | No data were excluded from the analysis. |
| Replication | RNA-seq and metabolite detection were performed with at least three replicates, and ATAC-seq with two replicates. |
| Randomization | Samples for the root development were collected from randomly selected plants exhibiting uniform growth conditions. |
| Blinding | Blinding was not required for this study. |

# Reporting for specific materials, systems and methods

We require information from authors about some types of materials, experimental systems and methods used in many studies. Here, indicate whether each material, system or method listed is relevant to your study. If you are not sure if a list item applies to your research, read the appropriate section before selecting a response.

## Materials & experimental systems

| n/a | Involved in the study |
| --- | --- |
| ☒ | ☐ Antibodies |
| ☒ | ☐ Eukaryotic cell lines |
| ☒ | ☐ Palaeontology and archaeology |
| ☒ | ☐ Animals and other organisms |
| ☒ | ☐ Clinical data |
| ☒ | ☐ Dual use research of concern |
| ☐ | ☒ Plants |

## Methods

| n/a | Involved in the study |
| --- | --- |
| ☒ | ☐ ChIP-seq |
| ☐ | ☒ Flow cytometry |
| ☒ | ☐ MRI-based neuroimaging |

# Plants

Seed stocks

Seeds were procured from its origin production area, Suining City (Sichuan, China), and were subsequently planted in the greenhouse of the Agricultural Genomics Institute at Shenzhen (Chinese Academy of Agricultural Sciences, China). Seeds were planted in March 2021. Sampling commenced in May 2021, with a monthly interval, resulting in a total of six sampling events. Another set of plants were planted in August 2020. Upon their flowering in April 2021, five distinct tissues were sampled, including roots, stems, mature leaves, young leaves, and flowers.

Novel plant genotypes

N/A

Authentication

N/A

# Flow Cytometry

## Plots

Confirm that:

☒ The axis labels state the marker and fluorochrome used (e.g. CD4-FITC).

☒ The axis scales are clearly visible. Include numbers along axes only for bottom left plot of group (a 'group' is an analysis of identical markers).

☒ All plots are contour plots with outliers or pseudocolor plots.

☒ A numerical value for number of cells or percentage (with statistics) is provided.

## Methodology

Sample preparation

Fresh leaves were vertically chopped with a disposable razor blade to release nuclei in cold LB01 lysis buffer. The nuclei suspension was filtered through a 40 µm cell strainer, stained with 20 µg mL-1 propidium iodide and 20 µg mL-1 RNase A, and ice-bathed for 30 min in the dark.

Instrument

CytoFLEX (Beckman Coulter, Miami, FL, USA)

Software

CytExpert v.2.3

Cell population abundance

N/A

Gating strategy

N/A

☒ Tick this box to confirm that a figure exemplifying the gating strategy is provided in the Supplementary Information.
